# Pro-Tumoral Inflammatory Myeloid Cells as Emerging Therapeutic Targets

**DOI:** 10.3390/ijms17111958

**Published:** 2016-11-23

**Authors:** Gabor J. Szebeni, Csaba Vizler, Lajos I. Nagy, Klara Kitajka, Laszlo G. Puskas

**Affiliations:** 1 Avidin Ltd., Also kikoto sor 11/D., H-6726 Szeged, Hungary; lajos@avidinbiotech.com (L.I.N.); laszlo@avidinbiotech.com or puskas.laszlo@brc.mta.hu (L.G.P.); 2 Synaptogenex Ltd., Őzsuta utca 20995/1, H-1037 Budapest, Hungary; 3 Department of Biochemistry, Biological Research Center, Hungarian Academy of Sciences, Temesvari krt. 62., H-6726 Szeged, Hungary; vizler.csaba@brc.mta.hu; 4 Department of Genetics, Biological Research Center, Hungarian Academy of Sciences, Temesvari krt. 62., H-6726 Szeged, Hungary; klarakitajka@gmail.com

**Keywords:** tumor-associated macrophages, myeloid-derived suppressor cells, inflammatory tumor microenvironment

## Abstract

Since the observation of Virchow, it has long been known that the tumor microenvironment constitutes the soil for the infiltration of inflammatory cells and for the release of inflammatory mediators. Under certain circumstances, inflammation remains unresolved and promotes cancer development. Here, we review some of these indisputable experimental and clinical evidences of cancer related smouldering inflammation. The most common myeloid infiltrate in solid tumors is composed of myeloid-derived suppressor cells (MDSCs) and tumor-associated macrophages (TAMs). These cells promote tumor growth by several mechanisms, including their inherent immunosuppressive activity, promotion of neoangiogenesis, mediation of epithelial-mesenchymal transition and alteration of cellular metabolism. The pro-tumoral functions of TAMs and MDSCs are further enhanced by their cross-talk offering a myriad of potential anti-cancer therapeutic targets. We highlight these main pro-tumoral mechanisms of myeloid cells and give a general overview of their phenotypical and functional diversity, offering examples of possible therapeutic targets. Pharmacological targeting of inflammatory cells and molecular mediators may result in therapies improving patient condition and prognosis. Here, we review experimental and clinical findings on cancer-related inflammation with a major focus on creating an inventory of current small molecule-based therapeutic interventions targeting cancer-related inflammatory cells: TAMs and MDSCs.

## 1. Introduction

In the first part of our review we summarize the current knowledge of the role of tumor-infiltrating immune cells in tumor pathogenesis. Briefly, while immune surveillance may eliminate malignant cells, thus preventing tumor formation in the early stage, in late stage cancers, several components of the immune system may promote, rather than suppress tumor growth. In the second part of the review we point out that, intentionally or unintentionally, many anti-tumor drugs target tumor-promoting myeloid-derived suppressor cells (MDSCs) and tumor-associated macrophages (TAMs). We also provide an extensive, although not exhaustive, list of these small molecule—based therapeutic agents and their targets. Synthesizing these data, rational strategies can be proposed for identifying new tumor therapies that more specifically target, eliminate or re-educate, tumor promoting myeloid and lymphoid cells.

## 2. Linking Inflammation and Cancer

Since the observation of Virchow in 1863, it has long been known that the tumor microenvironment constitutes the soil for the infiltration of inflammatory cells and for the release of inflammatory mediators [1]. Although the coordination of both innate and adaptive immune infiltrate with inflammatory mediators are rendered to serve the elimination of microbial invaders or malignant cells in concert with tissue repair and remodeling, under certain circumstances inflammation remains unresolved and smouldering which promotes cancer development. Now, it is estimated that due to unresolved inflammation 15%–20% of cancer deaths are related to chronic inflammation worldwide [2]. In a seminal study, Hanahan and Weinberg identified six hallmarks of cancer [3]. Due to the fact that inflammatory mediators can cause genetic instability, Mantovani and his colleagues proposed that cancer-related inflammation represents the seventh hallmark of cancer [4]. After a renewal of the seminal paper of Hanahan and Weinberg tumor promoting inflammation as a key hallmark was added to the complexity of cancer [5].

Inflammation sharing signal transduction networks of malignant transformations may arise from genetic defects and alterations in neoplastic cells as an intrinsic pathway [6]. On the other hand, inflammation predisposing for cancer can be driven extrinsically by infections (Helicobacter pylori, hepatitis) [7,8], autoimmune diseases (Crohn’s disease, ulcerative colitis) [9], chronic exposure to irritants (asbestos) [10,11] or by multiplex factors like in the case of prostatitis influenced by bacteria, diet and physical trauma to glandular epithelium by *corpora amylacea* and calculi [12].

In line with the above statements, several molecular evidences link unresolved inflammation and cancer. Here, we highlight molecular evidences of inflammation-driven cancer development or progression. Inflammatory mediators such as IL-1β promote angiogenesis [13] and overexpression of IL-1β mobilized myeloid-derived suppressor cells and induced gastric inflammation associated cancer [14]. IL-1β and TNF-α may alter stromal cells enhancing the expression of CCL2, CXCL8, and CCL5 by cancer-associated fibroblast and mesenchymal stem cells in the inflammatory tumor microenvironment of breast cancer [15]. TNF-α and IL-6 produced by the immune infiltrate and tumor cells are also considered as master switches between inflammation and cancer sustaining cellular transformation, survival, proliferation, angiogenesis, and metastasis [16,17]. IL-10 is considered as another arm of inflammation associated cancer since both mice and humans deficient in IL-10 developed malignancy [18,19], IL-10 was required for the physiological protective, anti-inflammatory effects of CD4+ CD25+ regulatory lymphocytes to interrupt colon carcinogenesis in mice [20]. The micro RNA, miR-155 may represent another molecular link between inflammation and cancer since elevated miR-155 level of inflammatory cells correlated with malignancy [21]. Carlo M. Croce and his colleagues reported that miR-155 down-regulated core mismatch repair proteins and increased the spontaneous mutation rate [22,23]. Under inflammatory conditions, reactive oxygen (ROS) and reactive nitrogen species (RNS) are released from macrophages, neutrophils and epithelial cells which could cause 8-nitroguanin mutagenic DNA lesions [24,25], moreover it was shown that myeloperoxidase catalyzed formation of hypochlorous acid (HOCl) was responsible for neutrophil induced genotoxicity in lung cancer [26]. Besides direct mutagenic roles of ROS or ROS-related molecular species, ROS as a signaling molecule can influence the expression of several cancer-related genes, including those affecting cell survival, angiogenesis, altered metabolism [27], and has great impact on T-cell immune response in cancer microenvironment [28].

Lifestyle has a great impact on human health. Due to adipose inflammation and metabolic dysfunction excess body weight contributes to obesity-related higher cancer incidence and mortality causing 14% and 20% cancer deaths in obese men and women above 50 years, respectively [29]. Reinforces the link between inflammation and cancer that pharmacological targeting of inflammatory cells and molecular mediators may establish therapies improving patient condition and prognosis. Long term use of non-steroid anti-inflammatory drugs (NSAID) as analgesics and antipyretics which are mostly nonselective cyclooxygenase inhibitors reduced incidence and mortality among others in esophageal adenocarcinoma, colorectal and stomach cancer [30,31].

The most common myeloid infiltrate in solid tumors is composed by myeloid-derived suppressor cells (MDSCs) and tumor-associated macrophages (TAMs). TAMs represent the major infiltrate of leukocytes in the tumor, a population of alternatively activated M2-like macrophages endowed with pro-tumoral functions such as: immunosuppression, promoting angiogenesis and cancer cell dissemination [32]. While classically activated, M1-like macrophages are pro-inflammatory (IL-12^high^, TNF-α^high^), phagocytic (MHCII^high^) and immunostimulatory expressing co-stimulatory molecules (CD40, CD80, CD86) and recruiting Th1 cells, M2 macrophages play a role in the resolution of inflammation, express anti-inflammatory molecules (IL-10, TGF-β, IL-1Ra), scavenger (CD163) and C-type lectin (CD206, CD301, dectin-1) receptors, recruit Th2 and regulatory T-cells (T-regs) [33]. MDSCs are CD11b+ and Gr1+ heterogeneous populations of immature myeloid cells developed from bone marrow common myeloid progenitors [34], MDSCs are precursors of granulocytes, monocytes, macrophages and dendritic cells. MDSCs are classified as Ly6C+ monocytic (M-MDSC) and Ly6G+ granulocytic (G-MDSC) subpopulations in mice [35]. Due to the lack of Gr1 homologue in humans the identification of MDSCs is not so evident, human MDSCs consist of phenotypically more heterogeneous population of myeloid cell precursors, briefly M-MDSC (CD11b+, HLA-DR^−/low^, CD33+, CD14+, CD15^−^), G-MDSC (CD11b+, HLA-DR^−/low^, CD33+, CD15+ or CD66b+) or the less well defined more immature MDSCs (CD14^−^, CD15^−^) [36,37]. These cells promote tumor growth by several mechanisms including their inherent immunosuppressive activity, promotion of neoangiogenesis, mediation of epithelial-mesenchymal transition and altering cancer cell metabolism. The pro-tumoral functions of TAMs and MDSCs are further enhanced by their cross-talk offering a myriad of potential anti-cancer therapeutic targets. Since TAMs and MDSCs among the cellular and molecular stromal constituents in the tumor microenvironment shape anti-tumor immunity and could be responsible for chemoresistance [38] we highlight the main pro-tumoral mechanisms of myeloid cells without a plenitude to give a general overview about their phenotypical and functional diversity representing examples of possible therapeutic targets. Our major focus is on the detailed review of small molecule-based therapeutic concepts targeting TAMs and MDSCs. Overall phenotypical and functional description of TAMs and MDSCs is reviewed elsewhere [39,40,41,42].

## 3. Pro-Tumoral Functions and Mediators of Inflammatory Myeloid Cells, as Potential Therapeutic Targets

### 3.1. Immunosuppression

TAMs and MDSCs promote immune escape inhibiting both adaptive and innate immunity through a variety of diverse mechanisms paralleled by declined T-cell functions with higher intensity in elderly [43,44]. Mainly G-MDSCs accumulate in peripheral lymphoid organs where they possess potent antigen specific suppressive activity, in contrast MDSCs are represented mainly by M-MDSCs in the tumor where they exert non antigen specific suppression and they rapidly differentiate toward TAMs [34]. Granulocytic MDSCs-derived ROS act in cell-cell contact manner, while monocytic MDSCs produce RNS and act through soluble mediators [45,46]. These radicals disrupt T cell receptor (TCR), IL-2 receptor signaling and MHC-TCR interactions [47,48]. MDSCs deplete arginine and cysteine, which are required for T-cell activation and proliferation. In addition, they secrete IL-10 and TGF-β, which down-regulate the Th1 driving cytokine IL-12 in macrophages [35]. MDSC-derived IL-10 and VEGF-A inhibit dendritic cell maturation. MDSCs promote the expansion and recruitment of both natural and induced T-regs, which further skew the tumor specific immune response into tolerance [43,48,49]. Human CD14+ peripheral monocytes can acquire MDSC-like phenotype suppressing autologous T-cell activation and IFN-γ production by melanoma produced cyclooxigenase-2 (COX-2) [50]. Subpopulations of MDSCs can give rise to CD11b+ F4/80+ macrophages with potent immunosuppressive properties [51]. Low oxygen supply via hypoxia-inducible factor-1α (HIF1-α) promotes MDSC differentiation into TAM in the tumor microenvironment [52].

In established tumors MDSC-, T-reg- or TAM-derived IL-10, found in high concentration in the tumor microenvironment, stimulate TAMs to convey inhibitory signals to T-cells through the expression of B7-H1 (PD-L1) [53] and B7-H4 [54]. It has been shown in renal cell carcinoma that TAMs produce substantial amount of CCL2 and immunosuppressive IL-10, in a 15-lipoxygenase-2-dependent way. TAMs also induce the pivotal regulatory T-cell transcription factor FOXP3 and the inhibitory cytotoxic T-lymphocyte antigen 4 (CTLA4) in T-cells, mediating immune tolerance [55]. TAMs further dampen tumoricidal CD8+ cytotoxic T-cell activation by arginase-1 (ArgI) which converts arginin to ornitin, leading to depletion of the key T-cell metabolite l-arginin [56]. TAMs display low MHCII expression, with poor antigen presenting capacity [57]. Myeloid cell specific ablation of adenosine A_2A_ receptor resulted in reduced melanoma tumor growth with significant increase in MHCII and IL-12 expression in TAMs with concomitant reduction of IL-10 expression in TAMs and MDCSs [58]. It has been reported that tissue resident alveolar macrophages underwent C5a dependent proliferation in a murine breast cancer model, these alveolar macrophages dampened tumor specific Th1 response and prevented the maturation of dendritic cells [59]. In a seminal study of Bronte and his colleagues it was reported that the peripheral tolerance to tumor antigens occurs in the spleen where CD11b+ Gr1^int^ Ly6C^high^ myeloid cells expand and tolarize memory CD8+ T-cells [60]. In a recent report pancreatic adenocarcinoma up-regulated factor (PAUF) not only enhanced the accumulation of MDSCs in the spleen but also increased the immunosuppressive phenotype of MDSCs by TLR4 dependent upregulation of arginase, nitric oxide (NO) and ROS [61]. Accumulating evidence supports that tumor or tumor stroma-derived free or microvesicle wrapped soluble mediators (IL-10, indolamine-2,3-deoxigenase, ROS, ArgI, PGE_2_) and even cell junction proximity with myeloid cells endow TAMs and MDSCSs with immunosuppressive phenotype dampening both innate and adaptive tumor cell clearance [44,62,63].

### 3.2. Angiogenesis

Most solid tumors remain dormant up to 1 mm^3^ volume even for decades. Their progression depends on sequential events like the angiogenic switch, an essential step in tumor progression to malignancy [64]. Tumor infiltrating myeloid cells are armed with an arsenal of angiogenic factors, which potentiate tumor invasiveness through the initiation of new blood or lymphatic vessels [43]. In a pioneer study it was proven that the angiogenic switch leading to new tumor vasculature was highly dependent on TAMs, as their genetic depletion diminished angiogenesis in PyMT oncogene driven breast cancer model [65]. In addition, the analysis of human specimens revealed a strong correlation between CD163 TAM infiltration and microvessel density in endometrioid carcinoma [66].

It has been reported that tumor or tumor stroma-derived G-CSF induced Bv8 expression in CD11b+ Gr1+ cells, which enhanced myeloid cell expansion in blood and tumors and increased tumor angiogenesis. Evidence suggests that blocking of Bv8 reduced myeloid infiltrate, angiogenesis and consequently tumor growth [67]. Melanoma derived CSF-1 stimulated macrophages to produce VEGF-A [68]. In renal cell carcinoma, VEGF level, microvessel density and high TAM infiltration have poor prognostic values, associated with high disease recurrence [69]. Amplification of inflammation, when LDL receptor-related protein (LRP1) was deleted in myeloid lineage cells, an increase in TAM density contributed to increased amount of VEGF and consequently higher vascularization in the microenvironment of pancreatic carcinoma [70]. Melanoma conditioned TAMs to produce adrenomedullin (ADM), which in turn mediated angiogenesis by both paracrine (endothelial nitric oxide synthase signaling) and autocrine (M2 polarization of TAMs) effects [71]. Semaphorin 4D (Sema4D) was also reported to be responsible for TAM mediated angiogenesis in a murine breast cancer model. Sema4D production in TAMs is activated by hypoxia (HIF1-α) and exerts its activity on endothelial cells through its receptor, plexin B1, activating the c-Met tyrosine kinase that promotes the production of a series of cytokines and proteases involved in angiogenesis and subsequent metastasis [72]. An elegant study added new molecular players to the complexity of TAM-mediated angiogenesis. Kale et al. delineated a model in which unknown soluble mediators from melanoma cells induced osteopontin (OPN) production by TAMs. Binding of autocrine OPN to the α9β1 integrin activated TAMs to produce more PGE_2_ and also augmented MMP-9 expression, to effectively regulate melanoma growth through angiogenesis and metastasis [73]. Furthermore, Wnt signaling plays an important role mediating TAM functions, especially in the context of tumor invasion and angiogenesis via TAM derived Wnt7b [74]. In a chick chorioallantoic membrane assay multiple myeloma derived G-MDSC exerted potent pro-angiogenic effect via up-regulation of a series of angiogenic factors, among others angiopoietin-1, angiopoietin-3, leptin, CCL3, PD-ECGF, and TIMP-4 [75].

The Tie-2 angiopoetin-2 receptor expressing monocytes (TEMs) represent the main monocyte population in tumors distinct from TAMs, with a profound angiogenic effect [76]. Angiopoetin-2 is released by tumor associated endothelia cells and is a potent chemoattractant for TEMs. Hypoxia upregulates both Tie-2 and angiopoetin-2 expression leading to the accumulation of TEMs [77].

### 3.3. Epithelial-Mesenchymal Transition (EMT), Matrix Remodeling, Metastasis

Epithelial-mesenchymal transition (EMT) refers to a functional and morphological change when an epithelial cell loses proximal adhesions, cell-cell junctions and acquires mesenchymal motile phenotype. Although EMT is a key process in tissue development and regeneration lots of data accumulated in the last decade about how under pathological circumstances EMT may contributes to malignancy during cancer microevolution. However, the role of EMT in cancer is not fully understood [78]. It has long been known that tumor infiltrating myeloid cells contribute to cancer dissemination causing fatal metastatic disease. In a spontaneous murine melanoma model CCL5 attracted MDSCs to the tumor where MDSCs promoted cancer cell dissemination by induction of EMT via TGF-β, EGF and HGF pathways [79]. It has been published that TAMs facilitated the EMT of pancreatic cancer cells, by upregulating the mesenchymal markers like vimentin, snail and inhibiting the epithelial marker E-cadherin [80]. Tumor induced MDSCs facilitated nasopharyngeal carcinoma lung metastases via induction of EMT in carcinoma cells via cell-cell contact. TGF-β and iNOS enhanced tumor COX-2 expression which activated the β-catenin/TCF4 pathway resulting in EMT in carcinoma cells [81]. In breast cancer model EMT triggered the release of soluble mediators (IL-6, IL-8, sICAM, PAI-1 and GM-CSF) which induced angiogenesis and recruited MDSCs which might favour cancer spread [82]. However, according to other groups EMT is not required for metastasis rather is responsible for chemoresistance of tumor cells [83,84]. Nevertheless, EMT contributes to the intra-tumor heterogeneity by promoting the stemness of cancer cells [85]. Cancer stem cells (CSCs) are a drug-resistant, low immunogenic highly hidden subpopulation within a solid tumor, moreover these CSCs are highly tumorigenic and invasive [85]. In an ovarian carcinoma model MDSCs triggered miR-101 expression in cancer cells, subsequently miR-101 silenced corepressor gene C-terminal binding protein-2 (CtBP2) which resulted in increased cancer stemness and dissemination [86]. Another microRNA, miR-126a released in exosomes of doxorubicin treated MDSCs promoted breast tumor lung metastasis through the induction of IL-13+ Th2 cells [87].

Hagemann et al. reported that co-culture of macrophages and tumor cells caused TNF-α-dependent activation of both JNKII and p65 NB-κB, which induced expression of extracellular matrix metalloprotease inducer (EMMPRIN) and macrophage migration inhibitory factor (MIF) in malignant cells, which further increased MMP secretion of macrophages [88]. A similar experimental concept led to the finding that macrophage-derived Wnt5 can activate AP-1/c-Jun in breast cancer cells, increasing their MMP-7 production [89]. In another study, macrophage-conditioned medium induced EMT and the invasiveness of hepatocarcinoma cells, which was dependent on c-Src-mediated induction of β-catenin phosphorylation, leading to destabilization of adherent junctions [90]. TAMs induced tumor cell migration and invasiveness also by Cox-2-dependent release of MMP-9 in human basal cell carcinoma [91].

Tissue resident macrophages of the liver, the Kupffer cells had a bimodal effect on colorectal cancer liver metastasis. Depletion of Kupffer cells before tumor induction resulted in increased tumor burden whereas late stage depletion of Kupffer cells decreased VEGF expressing infiltrates and increased CD3+ T-lymphocytes consequently diminishing liver tumor load [92]. A more detailed review about the metastatic effect of immune infiltrate has been extensively covered in other publications [93,94].

### 3.4. Altered Metabolism

Metabolic adaptation is a key phenomenon not only in tumor cells but also in the tumor stroma components. Hypoxia forces cells to shift their metabolism towards glycolysis, via upregulation of HIF-1α dependent genes, including the pyruvate kinase isoenzyme type M2, to produce ATP regardless the oxygen availability (‘Warburg effect’) [95]. Surprisingly IFN-γ and/or LPS activated M1 macrophages display an increased glycolytic flux, rapidly providing the energy required for their functions. In contrast, M2 macrophages exhibit enhanced fatty-acid oxidation and oxidative phosphorylation, with lower rate of glycolysis, sustaining their long-term activities [96]. In IL-4 polarized macrophages Signal Transducer and Activator of Transcription-6 (STAT6) induces the peroxisome proliferator-activated receptor gamma coactivator 1-beta (PGC-1β) transcriptional co-activator, which further promotes M2 polarization by induction of ArgI and enzymes involved in fatty-acid oxidation and mitochondrial oxidative phosphorylation [97]. During M2 polarization, the NAD^+^-dependent deacetylase Sirtuin-1 activates PGC-1β and inactivates p65 NF-κB, thus promoting the shift toward oxidative metabolism and alternative phenotype. TLR4 activation induces the Nicotinamide phosphoribosyltransferase (NAMPT) enzyme which produces NAD^+^, causing a negative feedback on macrophage activation [96,98]. Although both glycolytic and oxidative consumption rate were higher in tumor MDSCs compared to splenic MDSCs [34], Hossain et al. reported that tumor induced MDSCs increase fatty acid uptake and activate fatty acid oxidation as main metabolic programs [99]. Due to high glycolytic activity tumor cells enhance lactate production by elevated lactate dehydrogenase-A (LDH-A) expression. It has been shown that tumor cell specific LDH-A knockdown resulted in smaller tumors, decreased frequency of MDSCs accompanied with increased NK cytolytic function of NK cells in Pan02 pancreatic cancer model [100].

Epidemiologic studies have been published about the anti-cancer effects of polyunsaturated fatty acids (PUFAs) [101], on the other hand other reports link PUFAs with cancer risk and progression [102]. This discrepancy may rely on the difficulties to record dietary data accurately and also may rely on genetic variations in host PUFA metabolism [102]. Recently, we have showed the radiosensitizing role of PUFAs in human glioma cells [103]. It has been reported that PUFAs promote the expansion of MDSCs in the bone marrow, spleen and blood by activating the Janus kinase/Signal Transducer and Activator of Transcription-3 (JAK/STAT3) signaling. PUFA treatment augmented the T-cell suppressive function of MDSCs which was dependent on increased NADPH oxidase p47^phox^ and consequently elevated ROS production [104].

Macrophages play an important role in the clearance of senescent erythrocytes and the recycling of iron from hemoglobin. Alternatively activated macrophages upregulate the hemoglobin scavenger receptor CD163 (heme uptake) and the iron exporter Ferroportin [105], while classically activated macrophages favor iron retention by high Ferritin (iron storage) and low expression of CD163 and Ferroportin [106]. Thus, M2 macrophages are programmed for iron export to support tissue remodeling and proliferation, while M1 macrophages express bacteriostatic and tumoricidal activity [107].

MDSCs deplete amino acids essential for T-cell survival and functions (e.g., arginine) or tumor induced oxidative metabolism of MDSCs produce reactive oxygen species (e.g., H_2_O_2_) or reactive nitrogen intermediates (e.g., peroxinitrit, NO) [108]. However, we do not know much about their other metabolic programs other than the above mentioned immunosuppressive functions linked to metabolic activity of MDSCs [109].

## 4. Therapeutic Interventions Targeting TAMs and MDSCs, Tuning the Balance

Almost half of poorly-differentiated and 95% of anaplastic thyroid cancer cases showed high TAM infiltration, which correlated with poor survival rate [110]. Lymph node specimens of classic Hodgkin’s lymphoma showed high CD68+ macrophage infiltrate and gene expression profiling revealed a gene signature of TAMs associated with primary treatment failure and shortened survival [111]. In Ewing sarcoma patients, higher levels of CD68+ macrophages stimulating angiogenesis and osteoclastogenesis were associated with poorer overall survival [112]. In lung adenocarcinoma the majority of TAMs showed M2 polarization accompanied by more aggressive progression, lymphangiogenesis and lymph node metastasis [113]. In diffuse large B-cell lymphoma high CD68+ macrophage infiltration correlated with poor treatment outcome [114], and according to a meta-analysis the high density of TAMs was associated with worse overall survival in patients of breast, bladder, ovarian, gastric, and urogenital cancer [115]. Although there are reports about the positive effects of TAMs in colorectal cancer (CRC) [116,117], it was also shown that intra-tumoral TAMs in CRC correlated with depth invasion, lymph node metastasis and disease progression [118]. Another myeloid populations of tumor promoting cells are immature myeloid precursors, M-MDSCs and G-MDSCs. Several studies reported an elevated level of MDSCs in the blood of human cancer patients in melanoma, prostate cancer, bladder cancer, hepatocellular carcinoma (HCC), non-small cell lung cancer (NSCLC), chronic lymphocytic leukaemia (CLL), esophageal squamous cell carcinoma (ESCC), Hodgkin lymphoma, renal cell carcinoma (RCC), and in head and neck squamous cell carcinoma (HNSCC) [37]. Increased MDSC percentage was associated with higher risk of death in pancreatic, esophageal, gastric cancer and melanoma [119,120].

As a body of evidence from human clinical studies suggests how TAMs and MDSCs may facilitate tumor progression, novel therapies directed against myeloid infiltrate are emerging both in the clinic and preclinical research. Possible therapeutic approaches include: (a) inhibiting the recruitment and/or proliferation of monocytes/macrophages; (b) their selective ablation or (c) re-education to tumoricidal rather than tumor promoting functions; (d) differentiate immature myeloid cells or (e) pharmacologically inhibit their mediators responsible for pro-tumoral functions. Remarkably, modulation of MDSC and macrophage function is frequently an off-target effect of diverse drugs originally designed for other therapies.

### 4.1. Inhibition of the Recruitment and/or Proliferation of Tumor-Associated Macrophages (TAMs) and Myeloid-Derived Suppressor Cells (MDSCs)

Chemokines are key agents that attract macrophages to tumors. Inhibition of the monocyte chemoattractant protein MCP-1 (CCL2) with bindarit resulted in reduced tumor growth in human melanoma xenografts [121]. Bindarit enhanced expression of the NF-κB inhibitor IKB-α, modulating cancer cell proliferation in vitro and caused the impairment of tumor growth and metastasis formation with reduction in myeloid cell infiltration, in animal models of prostate and breast cancer [122]. Surprisingly anti-CCL2 monoclonal antibody treatment did not affect TAM recruitment but polarized TAMs to a more antitumor phenotype, where the tumor regression was CD8+ T-cell dependent in a murine NSCLC cancer model [123] (Table 1).

Macrophage colony stimulating factor M-CSF (CSF-1) is a potent monocyte/macrophage growth factor. Radiotherapy induced TAM and MDSC expansion in prostate cancer patients with an increase in M-CSF serum level. Mechanistic studies revealed that DNA damage-induced kinase ABL1 enhanced CSF-1 expression, while selective inhibition of its receptor kinase CSF1R (CD115) by GW2850 or PLX3397 inhibitors hampered TAM recruitment and suppressed tumor growth in murine prostate [124] and thyroid [125] cancer models. Moreover, blockade of CSF1/CSF1R signaling by GW2850 and PLX3397 CSF1R inhibitors or by anti-CSF-1 not only blocked TAM and M-MDSC recruitment, but also killed CD206^high^ TAMs and reprogrammed the remaining TAMs to support anti-tumor immune activities in murine ductal pancreatic adenocarcinoma [126]. When anti-CSF1 treatment was combined with anti-PD-1/anti-CTLA4 immunotherapy with gemcitabine chemotherapy they observed complete tumor regression in 30% of mice and an average tumor regression of 85% [126]. We showed that during cancer-driven granulo-monocytopoiesis colony stimulating factors (CSFs: G-CSF, GM-CSF, M-CSF) stimulate the expansion and recruitment of tumor promoting myeloid cells wherein retinoic-acid-related orphan receptor 1 (RORC1) drives cancer-related myelopoiesis in response to CSFs, antagonizing CSFs prevented cancer driven-myelopoiesis or the ablation of RORC1 hampered generation of TAMs and MDSCs in line with reduced MN/MCA1 tumor growth and lung metastasis [127] (Table 1).

Treatment of Ma-Mel-51 human melanoma cells by vemurafenib, a selective inhibitor of B-Raf kinase inhibited the release of soluble factors to generate M-MDSCs in vitro. Moreover, vemurafenib blocked the ability of malignant cells to recruit both M-MDSC and ArgI+ G-MDSCs in the blood of patients with advanced melanoma [128] (Table 1).

### 4.2. Selective Ablation, Depletion of TAMs and MDSCs

Removal of unwanted alternatively activated macrophages and immature myeloid cells offers a promising therapy. Anti-CD115 monoclonal antibody treatment successfully reduced tumor growth and prolonged survival of mice due to depletion of F4/80+ TAMs in the MMTV-PyMT murine breast cancer model [129,130]. Zoledronic acid (ZA) a bisphosphonate is used to treat bone damage in cancer patients, but it also has been reported to reduce the percentage of TAMs and to revert their polarization from M2 to M1 [131,132]. Selective ablation of TAMs using a tumor microenvironment-activated, legumain sensitive doxorubicin-based prodrug LEG-3, depleted TAMs, decreased circulating tumor cells and MDSCs in the spleen, with inhibition of breast tumor growth and metastasis formation [133,134]. Similar results were achieved using clodronate encapsulated liposomes for selective depletion of macrophages in human melanoma xenografts and in dogs with soft-tissue sarcoma [121,135]. A licensed and commercially available anticancer agent, trabectedin (Yondelis^®^), induced apoptosis in mononuclear phagocytes (TAMs, monocytes), in a caspase-8 dependent manner, leading to less tumor growth and angiogenesis [136,137] (Table 2).

It has been reported the nucleoside analog, conventional chemotherapeutical agent gemcitabine caused apoptosis and necrosis of splenocytes, selectively reduced the expansion of Gr1+/CD11b+ splenic MDSCs preserving CD4+ and CD8+ T-cells and that was accompanied by augmented antitumor activity of CD8+ T-cells and enhanced IFN-β gene delivery in murine mesothelioma [138]. In the 4T1 murine breast carcinoma early gemcitabine treatment also decreased MDSCs and improved T-cell proliferation and IFN-γ response [139]. A recently published new approach, lipid nanocapsules loaded with a lauroyl modified form of gemcitabine enhanced therapeutic efficacy, reduced tumor infiltrating and splenic M-MDSCs, attenuated tumor-associated immunosuppression in murine lymphoma and melanoma models [140]. Another classic chemotherapeutic agent, the pyrimidine analog 5-fluorouracil (5-FU) also has been shown to cause apoptosis and depletion of MDSCs with stronger efficacy over gemcitabine. Depletion of MDSCs by 5-FU promoted IFN-γ production and anti-tumor response without significant effect on dendritic cells, T-cells, B-cells and NK cells [141]. Bruchard et al. reported that gemcitabine and 5-FU induced not simply apoptosis of MDSCs but also the activation of the Nlrp3 inflammasome leading to the secretion of the inflammatory cytokine IL-1β and consequently the production of CD4+ T-cell-derived, tumor growth promoting IL-17. In line with this gemcitabine and 5-FU treatment should be combined with the inhibitors of Nlrp3 or IL-1β signaling [142]. Wang et al. recently published that MDSC depleting chemoterapeutics (gemcitabine and 5-FU) combined with adoptive immunotherapy using cytokine induced killer cell therapy increased 1-year survival rates of metastatic renal cell carcinoma and advanced pancreatic cancer patients [143]. Cisplatin a traditional chemotherapeutic agent depleted 50% of tumor infiltrating Gr1+/CD11b+ MDSCs without the impairment of T and B cell subsets, additionally cisplatin abrogated the immunosuppressive phenotype of the rest myeloid infiltrate in B16 melanoma model [144]. SAR131675, the inhibitor of VEGFR-3 exerted anti-tumoral activity in murine 4T1 model via reduction of the frequency of splenic Gr1+/CD11b+ cells and F4/80^high^ TAMs [145]. Targeting A20, a zinc-finger protein over-expressed in MDSCs by small interfering RNA resulted in caspase-3 and caspase-8 dependent apoptosis of MDSCs and increased tumor specific T-cell response, consequently reduced tumor growth in mice [146]. Myeloid cell depletion is able to enhance vaccine efficacy since immunization with TLR9 and NOD-2 containing microparticles followed by anti-CD11 treatment further delayed tumor progression in a mouse model of epithelial ovarian cancer [147]. Ibrutinib, an irreversible inhibitor of Bruton’s tyrosin kinase (BTK) and IL-2 inducible T-cell kinase (ITK) inhibited not only the generation of human MDSCs in vitro but also the recruitment of CD11b+/Gr1+ MDSCs in the tumor and spleen in murine breast cancer and melanoma models. Ibrutinib significantly enhanced the efficacy of the anti-PD-L1 immunotherapy via MDSC depletion which was dependent on BTK inhibition in mice [148] (Table 2).

Sunitinib, a receptor tyrosine kinase inhibitor decreased both HLA-DR^−^ CD33+ CD15+ and HLA-DR^−^ CD33+ CD15^−^ MDSCs in the blood of renal cell carcinoma patients which was associated with the reversal of Th1 response by enhanced production of T-cell IFN-γ and reduction in CD3+ CD4+ CD25^high^ Foxp3+ T-regs [149]. In another recent study sunitinib reduced non-classical CD33+ CD14+ CD16+ MDSCs in the blood of cancer patients by apoptosis and the rest of CD33+ CD14+ CD16+ MDSCs showed less pSTAT3, ArgI and less suppressive activity on T-cell proliferation. Moreover, sunitinib responders showed decreased T-reg population and sunitinib synergized with radiotherapy improving patient progression-free survival [150]. Administration of sunitinib in combination with immunotherapy (a viral vector based cancer vaccine) with or without irradiation could further increase its antitumoral activity via depleting circulating and intra-tumoral MDSCs and elevation of the level of antigen specific cytotoxic T lymphocytes in mice [151,152] (Table 2).

### 4.3. Re-Education of TAMs and MDSCs to Exert Anti-Tumor Functions

Re-educating tumor promoting myeloid cells, tuning the balance by alleviating their immunosuppressive effect offer a therapeutic strategy to improve cancer outcome [153]. In vivo IL-12 treatment altered TAM profile, promoting their pro-inflammatory activities from IL-10^high^, TGF-β^high^ to a TNF-α^high^ phenotype in a murine lung cancer [154]. It was shown in the IFN-α/βR^−/−^ genetic model that endogenously produced type I interferons suppress the generation of TAMs, which indicate local application of IFN-α/β as a potential therapeutic [155]. Inhibition of p50/p50 NF-κB nuclear translocation in TAMs [156], and the inhibition of IKKβ kinase reversed TAM phenotype from pro-tumoral to classically activated tumoricidal: IL-12^high^, MHCII^high^, IL-10^low^, Arginase-1^low^ [156,157].

Macrophage migration inhibitory factor (MIF) deficient macrophages showed decreased immunosuppressive and pro-angiogenic gene expression with less tumor burden in mice. Pharmacological targeting of MIF by a small molecule antagonist, 4-iodo-6-phenylpyrimidine (4-IPP) also reduced tumor growth by reduction of ArgI and elevation of TNF-α expression in TAM, furthermore 4-IPP attenuated TAM and both splenic Gr1^high^ Ly6G+ G-MDSC and Gr1^dim^, Ly6G^−^ M-MDSCs mediated immunosuppression [158]. In a male-predominant hepatocellular carcinoma model, 17β-estradiol (E2) repressed alternative macrophage activation and tumor growth through the inhibition of Janus activated kinase-1 (JAK1) and STAT6 phosphorylation [159]. The fact that M2 macrophages express higher legumain (a cysteine protease) on their cell surface allows selective therapy. Legumain targeted nanoparticles encapsulating hydrazinocurcumin suppressed STAT3 and re-educated TAMs, to be IL-12^high^, IL-10^low^ and TGF-β^low^, which resulted in suppression of tumor growth, metastasis and angiogenesis in vivo [160]. Several new curcumin derivatives have been synthesized and were confirmed to have anticancer activities, however their potential effects on TAMs and MDSCs would be interesting to test [161]. Another inhibitor of JAK1 and STAT3, a synthetic triterpenoid, bardoxolone methyl (C-28 methyl ester of 2-cyano-3,12-dioxooleana-1,9,-dien-28-oic acid, also known as CDDO-Me) abrogated the immune suppressive effect of MDSCs on CD8+ cytotoxic T-cells resulting in decreased tumor growth in mice [162,163]. 5,6-Dimethylxanthenone-4-acetic-acid (DMXAA, Vadimezan or ASA404) augmented tumor immunotherapy by increasing the infiltration of neutrophils and M1 macrophages in concert with the higher frequency of CD8+ T-cell recruitment to the tumor. The beneficial effect of DMXAA relied on the modulation of macrophages since the clodronate depletion of macrophages markedly alleviated the therapeutic response of DMXAA in mice [164]. Administration of yeast-derived whole β-glucan particles (WGP), a ligand of C-type lectin dectin-1, decreased tumor growth of Lewis lung carcinoma and E0771 mammary carcinoma in mice. WGP subverted the immunosuppression of both splenic and tumor MDSCs, reduced accumulation of G-MDSC and differentiated M-MDSC into CD11c+ professional antigen presenting cells successfully promoting Th1 differentiation and antigen cross-presentation to CD8+ effector cells [165,166]. Inhibitors (sildenafil, tadalafil, vardenafil) of phosphodiestarase-5 (PDE-5) by preventing the hydrolysis of cGMP have been used to treat erectile dysfunction, pulmonary arterial hypertension and cardiac hypertrophy in the clinical practice [167,168]. Serafini et al. showed that sildenafil increased cGMP, reduced IL-4Rα expression and down-regulated ArgI and NOS2 enzymatic activity of tumor infiltrating MDSCs thereby reduced the immunosuppression of G1+/CD11+ myeloid cells with improving the efficacy of adoptive T-cell therapy in tumor bearing mice [169] (Table 3).

Immunotherapy boosts antigen specific anti-tumor immune response or augments the overall immune response by adjuvant therapy to modulate tumor microenvironment and combat different points of tumor-driven immune-escape mechanisms. Anti-phosphatidylserine antibody (2aG4) in combination with docetaxel inhibited the growth of LNCaP and PC3 human prostate xenograft models in SCID mice via repolarization of M2 TAMs to M1 with higher expression of TNF-α, IL-12, MHCII and elevated expression of co-stimulatory CD40, CD80, CD86 molecules. Docetaxel increased phosphatidylserine exposure on tumor vessels which were disrupted by these predominantly M1-like TAMs. Furthermore, 2aG4 decreased the infiltration of Gr1+ cells and differentiated MDSCs toward M1 macrophages and dendritic cells changing the microenvironment to immunostimulatory [170]. In human melanoma patients Ipilimumab (anti-CTLA4) therapy was the first one to improve patient survival at stage III/IV significantly reducing G-MDSC frequency followed by the reduction of ArgI producing CD3^−^ cells [171]. Although the frequency of M-MDSCs did not change by Ipilimumab treatment and M-MDSC level linearly correlated with the clinical outcome as a prognostic marker [172], 2-year survival probability after ipilimumab initiation was 34.5% for 99 patients with Lin^−^ CD14+ HLA-DR^low^ MDSC frequencies <5.1%, while there were no survivors among 65 patients with higher MDSC levels [173]. Blocking PD-1/PDL-1 immune checkpoint molecules by anti-PD or anti-PD-L1 antibodies in combination with GVAX, FVAX immunotherapy (GM-CSF or FLT3 expressing irradiated tumor cells) alone or followed by a-4-1BB stimulation or TLR9 agonist (CpG 1668) resulted in the rejection of 50% (GVAX-aPD-1 or FVAX-aPD-1) or 75% (GVAX-4-1BB-aPD-L1 or FVAX-4-1BB-aPD-L1) of ID8 ovarian carcinoma tumors in mice. Anti-PD-L1 decreased ArgI activity of MDSCs, furthermore GVAX-4-1BB-aPD-L1 or FVAX-4-1BB-aPD-L1 combinatorial immunotherapy restored T-cell immunity with increased IFN-γ and TNF-α production in concert with the elevation of T:MDSC cell ratio [174]. Polyclonal and poly-specific intravenous immunoglobulins (IVIgs), prepared from the plasma of thousands of human healthy donors repolarized human M2 macrophages toward M1 via FcγRIII (CD16) and Syk phosphorylation dependent manner, moreover IVIgs inhibited MC38 colon cancer progression which was dependent on macrophages, FcgRIII, FcgRIV and FcRg-chain [175]. Cationic dextran and polyethyleneimine repolarized MDSCs of 4T1 tumor bearing mice into anti-tumor cells to express tumoricidal cytokines (IL-12, TNF-α) with less production of immunosuppressive factors (IL-10, TGF-β) reactivating T-cell functions which resulted in reduced tumor growth and prolonged survival [176].

Adjuvants to augment cancer immunotherapy and overcome MDSC mediated immunosuppression provide immunostimulatory signals boosting the immune response via bacterial products or Toll-like receptors, cytokines and growth factors or by immunostimulatory delivery systems (e.g., nanoparticles targeting TAMs to deliver tumor antigens) [177,178]. Toll-like receptor-9 (TLR9) agonist CpG oligonucleotids (ODNs) affected on TLR9 expressing M-MDSC cells. CpG ODNs reduced intra-tumoral M-MDSC infiltration, NO and ArgI production, increased IL-12 expression of splenic M-MDSC losing their ability to suppress CD8+ T-cells. CpG ODNs induced the differentiation of M-MDSCs to F4/80+ macrophages supporting tumor elimination [179]. RNA adjuvant therapy mimicking dsRNA by Poly(I:C) modulated tumor infiltrating myeloid cells via TLR3/TICAM-1 pathway from tumor-supportive to tumor-suppressive [180]. Another adjuvant, co-administration of the TLR7 agonist imiquimod led to improved antitumor effect of cancer vaccine augmenting tumor specific immune response based on the decline of tumor infiltrating MDSCs and on the activation of antitumor NK 1.1+ and F4/80+ macrophages [181]. Tasquinimod, a novel antitumor agent has been reported to prolong the progression-free survival of human castration-resistant prostate cancer patients [182]. Tasquinimod enhanced the effectiveness of immunotherapy, inhibited the accumulation of Ly6C+ MDSCs and CD206+ M2-like TAMs via targeting S100A9, furthermore CD11b+ myeloid cells showed less ArgI and iNOS expression which resulted in significantly reduced tumor growth in murine models of prostate cancer and melanoma [183] (Table 3).

Although tumor infiltrating dendritic cells (CD45+ CD11c+ MHCII+) are not macrophages, they may arise from the same monocytic precursors and may share a series of signal transduction pathways leading to alternative activation [184]. These tumor-associated dendritic cells were transformed from immunosuppressive to highly immunostimulatory cells, capable to trigger a potent antitumor immune response by the administration of miR-155 mimetics, which inhibited CEBP/β, SOCS1, PU.1 transcription factors, leading to upregulation of TNF-α, IL-12 and IFN-γ in the tumor microenvironment [185].

Infiltration of myeloid cells to the tumor microenvironment is often associated with increased neoangiogenesis characterized by higher microvessel density in the tumor [186]. Inhibition of PI3Kγ and δ in myeloid cells by the small-molecule inhibitor IPI145 enhanced the efficacy of VEGF/VEGFR blockade anti-angiostatic therapy by sorafenib. IPI145 decreased intratumoral TAM, Gr1+ monocytes and tumor-associated neutrophils, moreover IPI145 induced perforin expression of cytotoxic T lymphocytes generating an immune stimulatory tumor microenvironment [187]. Prokineticin 2 (PK2 or Bv8) has been reported to play a role in the mobilization of myeloid cells and in the recruitment of TAMs and neutrophils to the tumor site promoting angiogenesis. A small molecule PK2 antagonist, PKRA7 inhibited tumor growth via interfering neovascularization of glioma and myeloid cell infiltration of pancreatic cancer xenografts, respectively [188] (Table 3).

Based on our current knowledge about the role of infiltrating immune cells in tumor growth, it seems plausible that alternatively activated macrophages might be among the main targets of conventional anti-tumor radiotherapy and chemotherapy as well. Radiotherapy and chemotherapy are known to damage the gut epithelium, facilitating the translocation of bacteria and contact of bacterial danger signals with the circulation [189,190]. Gut bacteria or their cell wall components were shown to induce a Type 1 macrophage polarization [191,192]. In addition, chemotherapeutic agents have a well-known immunosuppressive effect; in fact, some anti-tumor compounds, e.g., alkylating agents and antimetabolites, are also used for immunosuppression in transplantation or autoimmunity [193]. Immunosuppression, in turn, may also facilitate opportunistic infections that may lead to M1 type macrophage polarization. In accordance with these data, infections might indeed be associated with spontaneous tumor regression [194]. The hypothesis is further supported by observations that gut flora is crucial for an effective chemotherapy [195].

### 4.4. Differentiation of MDSCs

Since MDSCs represent immature myeloid cells with inherent immunosuppressive activity differentiation of MDSCs into mature myeloid cells thereby restoration of T-cell immunity would be a promising therapeutic strategy [196].

It was published quite early that pretreatment with TGF-β of human promyelocytic cells followed by 1,25-dihydroxyvitamin D3 (vitamin D3) treatment induced monocytic maturation [199], while in other study vitamin D3 treatment of mice having Lewis lung carcinoma reduced the frequency of myeloid progenitors and tumor-driven myelopoiesis associated immunosuppression leading to transient tumor regression and prominent metastasis reduction [200]. In a human phase 1B clinical study 25-dihydroxyvitamin D3 reduced the number of CD34+ immunosuppressive cells, increased HLA-DR expression, elevated plasma IL-12 and IFN-γ level in the blood of HNSSC patients [201]. Another vitamin D derivative, all-*trans* retinoic acid (ATRA) combined with GM-CSF differentiated immature myeloid Gr1+ cells, eliminated their inhibitory potential and restored the number of IFN-γ producing cells [202]. ATRA (>150 ng/mL in the blood) dramatically reduced the percentage of immature myeloid suppressive cells in the blood of human metastatic renal cell carcinoma patients and improved antigen specific T-cell response [203]. The TLR7/8 agonist imidazoquinoline-like molecule, resiquimod treated MDSCs differentiated to F4/80+ macrophages and CD11c+/MHCII+ (I-A^d+^) dendritic cells exerting potent T-cell stimulatory function [204] (Table 4.).

### 4.5. Pharmacological Targeting of the Pro-Tumoral Mediators of TAMs and MDSCs

Celecoxib a cyclooxigenase II (COX-2) inhibitor reverted TAM phenotype from M2 to M1, associated with reduced intestinal tumor progression [205]. Another COX-2 inhibitor, etodolac blocked M2 macrophage differentiation and suppressed metastasis formation in a murine breast cancer model [206]. MDSC induction from healthy human monocytes and their immunosuppressive phenotype induced by early-passage melanoma cells via cell-cell contact or close proximity was completely abolished by the COX-2 inhibitor celecoxib. Moreover, inhibition of STAT3 phosphorylation by Tyrphostin AG490 in patient-derived CD14+ cells alleviated their T-cell inhibitory function [50] (Table 5).

Many of the immunosuppressive effects of MDSCs rely on the release of ROS. Withaferin A (WA), a component of the root extract of *Withania somnifera* inhibited ROS production of Gr1+ CD11b+ MDSCs by inhibition of STAT3 phosphorylation, WA also reduced IL-10 production generated by MDSC-macrophage cross-talk. Macrophage secretion of pro-inflammatory cytokines IL-6 and TNF-α, which increase MDSC accumulation, was also reduced by WA, additionally WA delayed tumor progression with reduction of the accumulation of G-MDSCs in 4T1 mammary carcinoma bearing mice [207] (Table 5).

MDSCs inhibit T-cell proliferation not only by arginase, ROS, RNS and IL-10 but also by depleting cysteine. T-cells import cysteine by the ASC neutral amino acid transporter. Both APC cells and MDSCs express X_c_^−^ transporter for the uptake of cystine and APC cells export cysteine for T-cells. In contrast, MDSCs compete with APCs for the uptake of cystine and do not export cysteine. Therefore, MDSCs consume cystine and deprive T-cells from cysteine constraining T-cell activation and proliferation. *N*-acetyl cysteine (NAC) enters cells via ASC transporters, and is hydrolyzed rapidly to cysteine, restoring both CD4+ and CD8+ T-cell proliferation and activation [208] (Table 5).

Another component of amino acid metabolism, ornithine-decarboxylase (ODC) was showed to be a potential therapeutic target which is upregulated in Gr1+/CD11b+ MDSCs of tumor bearing mice. Inhibition of ODC by α-difluoromethylornithine (DFMO) decreased ArgI production in MDSCs consequently DFMO treated MDSCs failed to retain their suppressive activity which led to slower tumor growth in wild type mice but not in Rag1^−/−^ immunodeficient mice suggesting that DFMO treatment augments antitumor immunity via modulation of ODC in MDSCs [209] (Table 5).

## 5. Conclusions

**Figure 1:**
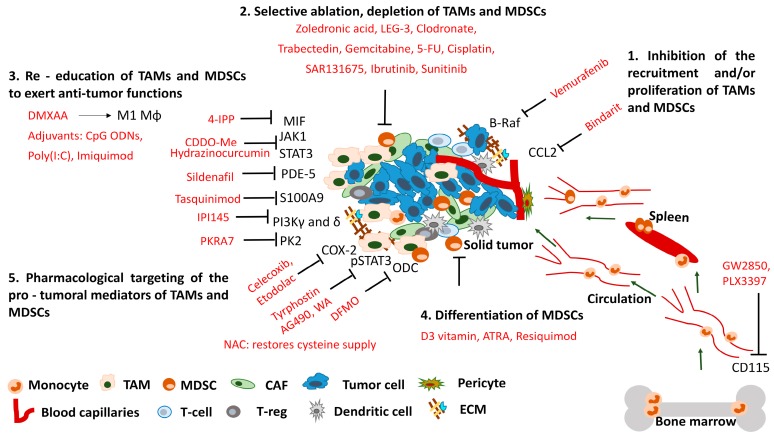
Small molecule-based therapeutic strategies to target TAMs and MDSCs in the tumor microenvironment. Solid tumor microenvironment constitutes a variety of cellular (MDSC, TAM, CAF, T-reg) and molecular stromal components (ECM) which hamper anti-tumor therapeutic response. We summarize current small molecule therapeutics (red) targeting TAMs and MDSCs. Possible therapeutic approaches include: (1) inhibition the recruitment and/or proliferation of monocytes/macrophages; (2) their selective ablation or (3) re-education to tumoricidal rather than tumor promoting; (4) differentiate immature myeloid cells or (5) pharmacologically inhibit their mediators responsible for pro-tumoral functions. Remarkably, modulation of MDSC and macrophage function is frequently an off-target effect of diverse drugs originally designed for other therapies. TAM: tumor-associated macrophage; MDSC: myeloid-derived suppressor cell; CAF: cancer-associated fibroblast; T-reg: regulatory T cell, ECM: extracellular matrix. Arrows refer to the direction of cell migration or stimulation; T-bar arrows refer to inhibition.

We have seen that many of the currently developed anti-cancer therapeutics and traditional chemotherapeutic agents target TAMs and MDSCs (Figure 1), augmenting the anti-tumor immune response and improving patient outcomes. Exploitation of these non-conventional immunomodulating effects might require different drug dosage or administration, as compared with those required for the primary indications of the agents.

Since the authorization and introduction of new clinical applications of already approved drugs is much safer, shorter, cheaper and faster, it is advisable to screen for TAM and MDSC targeting compounds from the FDA approved drug library. Developing and adopting both in vitro and in vivo assays for high throughput screening campaigns to identify compounds, which (1) inhibit the recruitment or proliferation of TAMs and MDSCs; (2) deplete or (3) reprogram them by reverting their tumor promoting phenotype to anti-tumor effectors, and/or (4) differentiate immature myeloid cells; and finally (5) pharmacologically block their pro-tumoral mediators, are of high importance.

Several pathomechanisms such as immunosuppression, angiogenesis, metastases and altered metabolism link chronic inflammation and cancer progression to worsened patient condition. On the other hand, the anti-tumor effect of a diverse array of pharmacological interventions converges on inhibition or re-education of alternatively activated tumor infiltrating immune cells. Hereafter intensive research should be conducted to reveal in depth the molecular players of chronic inflammatory conditions involved in cancer development or in the establishment of tumor microenvironment in order to identify potential targets of anti-cancer therapeutic interventions.

## Figures and Tables

**Table 1:** Chemical agents for the inhibition of the recruitment and/or proliferation of myeloid-derived suppressor cells (MDSCs) and tumor-associated macrophages (TAMs).

Compounds	Chemical Structures	In Vivo Effect	Mechanism of Action	References
Bindarit	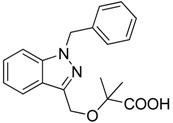	Decreases the infiltration of TAMs and MDSCs	Inhibits the synthesis of C-C motif chemokine ligand 2 (CCL2)	[121,122]
GW2850	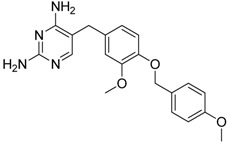	Decreases the infiltration of TAMs and MDSCs	Selective receptor kinase CSF1R (CD115) inhibitor	[124,125,126]
PLX3397 (Pexidartinib)	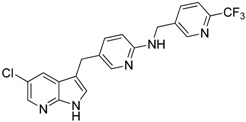	Decreases the infiltration of TAMs and MDSCs	Selective receptor kinase CSF1R (CD115) inhibitor	[124,126]
Vemurafenib	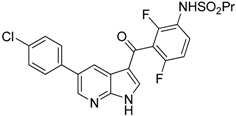	Blocks the recruitment of both M-MDSCs and G-MDSCs	Selective B-Raf kinase inhibitor	[128]

**Table 2:** Chemical agents for the selective ablation, depletion of TAMs and MDSCs.

Compounds	Chemical Structures	In Vivo Effect	Mechanism of Action	References
Zoledronic acid	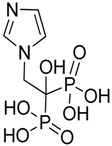	Reduced the number of TAMs and reverted their polarization from M2 to M1	Inhibits the active site of the enzyme farnesyl pyrophosphate (FPP) synthase in the mevalonate (Mev) pathway	[131,132]
Doxorubicin-based prodrug (LEG-3)	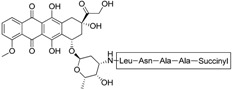	Depletes TAMs	LEG-3 is a legumain, an asparagynil endopeptidase activated prodrug. Doxorubicin is a DNA intercalator	[133,134]
Clodronate (encapsulated liposomes)	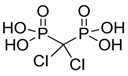	Depletes TAMs	Clodronate is converted to non-hydrolyzable ATP analogue intracellularly	[121,135]
Trabectedin (Yondelis^®^)	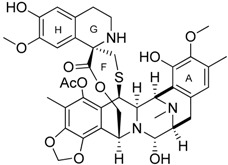	Induces apoptosis of mononuclear phagocytes (TAMs, monocytes)	Caspase-8 activation via TRAIL-Rs pathway	[136,137]
Gemcitabine	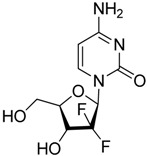	Reduces the expansion of Gr1+/CD11b+ splenic MDSCs	Nucleoside analog	[139]
5-fluorouracil (5-FU)	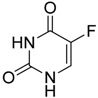	Causes apoptosis and depletion of MDSCs	Pyrimidine analog	[141]
Cisplatin	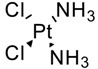	Depleted 50% of tumor infiltrating Gr1+/CD11b+ MDSCs	Forms DNA adducts	[144]
SAR131675	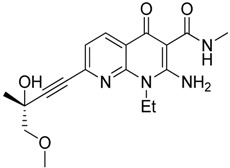	Reduces the number of splenic Gr1+/CD11b+ cells and F4/80^high^ TAMs	VEGFR-3 inhibitor	[145]
Ibrutinib	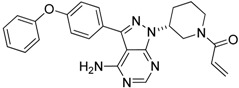	Inhibits the recruitment of CD11b+/Gr1+ MDSCs in the tumor and spleen	Irreversible inhibitor of Bruton’s tyrosin kinase (BTK) and IL-2 inducible T-cell kinase (ITK)	[148]
Sunitinib	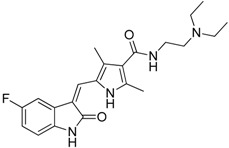	Reduces MDSCs in the blood, enhances IFN-γ+ Th1 response and reduces T-regs	Multi-targeted receptor tyrosine kinase inhibitor	[149,150,151,152]

**Table 3:** Chemical agents for the re-education of TAMs and MDSCs to exert anti-tumor functions.

Compounds	Chemical Structures	In Vivo Effect	Mechanism of Action	References
4-iodo-6-phenylpyrimidine (4-IPP)	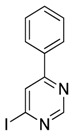	Reduces ArgI and elevates TNF-α expression in TAM, attenuates TAM and both splenic Gr1^high^ Ly6G+ G-MDSC and Gr1^dim^, Ly6G^−^ M-MDSCs mediated immunosuppression	Migration inhibitory factor (MIF) antagonist	[158]
Hydrazinocurcumin	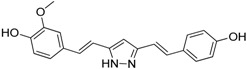	Re-educates TAMs to be IL-12^high^, IL-10^low^ and TGF-β^low^	Suppresses STAT3	[160]
Bardoxolone methyl (CDDO-Me)	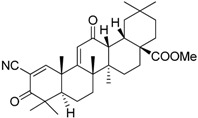	Abrogates the immune suppressive effect of MDSCs	JAK1 and STAT3 inhibitor	[162,163,196]
5,6 Dimethylxanthenone-4-acetic-acid (DMXAA, Vadimezan or ASA404)	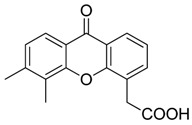	Increases the influx of neutrophils and anti-tumour (M1) macrophages to the tumour, induces macrophage activation, augments the therapeutic effects of immunotherapy	‘Stimulator of interferon gene’ (STING) agonist, multi-kinase inhibitor	[164,197]
Sildenafil (Viagra^®^)	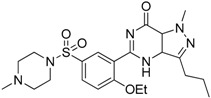	Down-regulates ArgI and NOS2 enzymatic activity of tumor infiltrating MDSCs	Phosphodiesterase-5 (PDE-5) inhibitor	[169]
Imiquimod	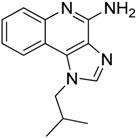	Decreases tumor infiltrating MDSCs and activates antitumor NK 1.1+ cells and F4/80+ macrophages in combination with immunotherapy	TLR7 agonist	[177,181]
Tasquinimod	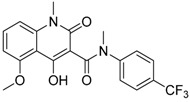	Inhibits the accumulation of Ly6C+ MDSCs and CD206+ M2-like TAMs	Orally active S100A9 inhibitor	[182,183,198]
IPI145 (Duvelisib)	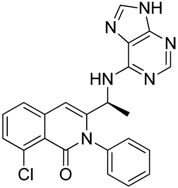	Enhances the efficacy of VEGF/VEGFR blockade anti-angiostatic therapy by sorafenib. IPI145 decreases intra-tumoral TAM, Gr1+ monocytes and tumor-associated neutrophils	Phosphatidylinositol-3 kinase γ and δ (PI3Kγ and δ) inhibitor	[187]
PKRA7	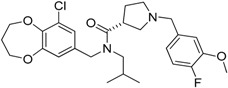	Inhibits the neovascularization of glioma and myeloid cell infiltration of pancreatic cancer	Prokineticin 2 (PK2 or Bv8) antagonist	[188]

**Table 4:** Chemical agents for the differentiation of MDSCs.

Compounds	Chemical Structures	In Vivo Effect	Mechanism of Action	References
D3 vitamin (Cholecalciferol)	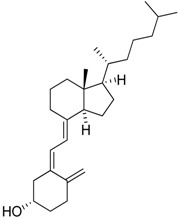	Induces monocytic differentiation, reduces tumor-induced myelopoiesis, reduces the number of CD34+ immunosuppressive cells	Calcitriol (vitamin D) receptor agonist	[199,200,201]
ATRA (Tretinoin)	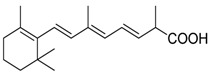	Combined with GM-CSF differentiates immature myeloid Gr1+ cells, eliminates their inhibitory potential	Retinoic acid receptor agonist	[202,203]
Resiquimod	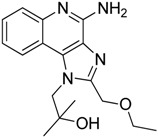	Differentiates MDSCs to F4/80+ macrophages	TLR7/8 agonist	[177,204]

**Table 5:** Pharmacological targeting of the pro-tumoral mediators of TAMs and MDSCs.

Compounds	Chemical Structures	In Vivo Effect	Mechanism of Action	References
Celecoxib	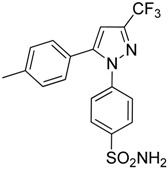	Reverted TAM phenotype from M2 to M1	Cyclooxygenase II (COX-2) inhibitor	[50,205]
Etodolac	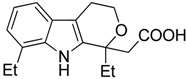	Blocks M2 macrophage differentiation and suppresses metastasis formation	COX-2 inhibitor	[206]
Tyrphostin AG490	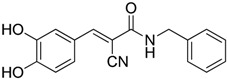	Decreases the T-cell inhibitory function of melanoma patient-derived CD14+ cells	Inhibits STAT3 phosphorylation	[63]
Withaferin A	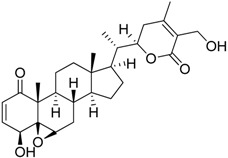	Reduces IL-10 production of MDSCs and the accumulation of G-MDSCs	Inhibits ROS production via inhibition of STAT3 phosphorylation	[207]
*N*-acetyl cysteine (NAC)	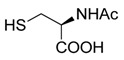	Restores both CD4+ and CD8+ T-cell proliferation and activation	Antioxidant and enters cells via ASC transporters, rapidly hydrolyzes to cysteine	[208]
α-Difluoromethylornithine (DFMO)	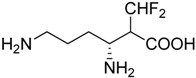	Decreases ArgI production in MDSCs	Ornithine-decarboxylase (ODC) inhibitor	[209]
